# Numerical Assessment of Electric Underfloor Heating Enhanced by Photovoltaic Integration

**DOI:** 10.3390/s25185916

**Published:** 2025-09-22

**Authors:** Hana Charvátová, Aleš Procházka, Martin Zálešák, Vladimír Mařík

**Affiliations:** 1 Faculty of Applied Informatics, Tomas Bata University in Zlín, 760 01 Zlín, Czech Republic; zalesak@utb.cz; 2 Department of Mathematics, Informatics and Cybernetics, University of Chemistry and Technology in Prague, 160 00 Prague, Czech Republic; a.prochazka@ieee.org; 3 Czech Institute of Informatics, Robotics and Cybernetics, Czech Technical University in Prague, 160 00 Prague, Czech Republic; vladimir.marik@cvut.cz

**Keywords:** underfloor heating, photovoltaic panels, hybrid heating systems, thermal efficiency, numerical simulation, integration of renewable energy, room air temperature, heat loss analysis, energy supply, thermal response time

## Abstract

The integration of electric underfloor heating systems with photovoltaic (PV) panels presents a promising approach to enhance thermal efficiency and energy sustainability in residential heating. This study investigates the performance of such hybrid systems under different energy supply scenarios. Numerical modeling and simulations were employed to evaluate underfloor heating performance using three electricity sources: standard electric supply, solar-generated energy, and a combined configuration. Solar irradiance sensors were utilized to collect input solar radiation data, which served as a critical parameter for numerical modeling and simulations. The set outdoor air temperature used in the analysis represents an average value calculated from data measured by environmental sensors at the location of the building during the monitored period. Key metrics included indoor air temperature, time to thermal stability, and heat loss relative to outdoor conditions. The combined electric and solar-powered system demonstrated thermal efficiency, improving indoor air temperature by up to 63.6% compared to an unheated room and achieving thermal stability within 22 h. Solar-only configuration showed moderate improvements. Heat loss analysis revealed a strong correlation with indoor–outdoor temperature differentials. Hybrid underfloor heating systems integrating PV panels significantly enhance indoor thermal comfort and energy efficiency. These findings support the adoption of renewable energy technologies in residential heating, contributing to sustainable energy transitions.

## 1. Introduction

Nowadays, energy efficiency methods and sustainable development are emerging as key concerns not only for industry professionals but also for households and construction stakeholders. In this regard, the integration of modern heating systems and electricity generation technologies marks a significant step toward energy independence and environmental responsibility [1]. It provides flexibility in energy management and economic advantages [2,3,4].

Underfloor heating stands out among residential heating methods. It works by warming the room directly through the large surface area of the floor. These systems gently heat the solid floor, which then transfers warmth upwards via a combination of conduction, radiation, and a touch of convection. By heating the floor itself, the warmth is distributed evenly throughout the room. A heated floor emits infrared radiation, warming people and objects directly rather than just heating the air, resulting in a more natural and comfortable experience [5,6].

When compared to traditional heating methods, underfloor heating and conventional systems each have their own benefits and drawbacks. Underfloor heating systems are very suitable for net-zero buildings because they operate at lower temperatures than forced-air systems, fit well with renewable sources like solar panels and heat pumps, making them more energy efficient [7,8]. They also provide consistent warmth from the ground up, eliminating cold spots, while traditional systems often lead to uneven heating.

Because underfloor heating does not rely on air circulation, it reduces the spread of dust and allergens; in contrast, forced-air systems may recirculate airborne particles. Radiant systems also help maintain natural humidity levels, whereas conventional methods can dry out indoor air. Underfloor heating is particularly suited for new construction but may require floor height modifications for retrofits. Traditional systems like forced-air units, on the other hand, are easier to install in existing buildings. Finally, a notable benefit of underfloor heating systems is their visual subtlety and aesthetic appeal. Electric systems use thin heating elements—such as cables, mats, or films—embedded beneath the flooring. When an electric current passes through them, Joule heating occurs. In this process, electrical energy is converted into heat through resistance, and this heat spreads evenly across the floor surface.

In terms of energy efficiency, radiant underfloor heating systems can achieve significant energy savings over conventional methods, some studies showing reductions in consumption of up to 34.75% [9]. The performance of underground heat exchangers and the mixing ratios of water flow are vital to maximize energy efficiency in these systems [10]. Likewise, the choice of floor materials influences both energy savings and temperature distribution, with some materials providing more uniform heating [11].

Optimized control strategies are essential to balance comfort with efficiency. These consider factors such as indoor air temperature and humidity [12]. However, some disadvantages include an added demand for the electrical system and higher upfront costs.

The basic assessment of subjective properties of subfloor heating systems can be performed both experimentally [13,14,15] and using software calculation tools [16,17,18,19,20]. The computer modeling of floor heating focuses on the issues of thermal comfort assessment, energy efficiency in heating systems, and radiant heating technologies. The key parameters in the modeling of the underfloor heating system include thermal comfort factors [14,21], energy efficiency considerations [22,23], and control strategies [24,25]. These parameters are essential for optimizing radiant underfloor heating systems’ performance and user satisfaction.

This study investigates the potential for combining electric underfloor heating with photovoltaic (PV) panels. This approach merges the comfort of modern heating with the economic and ecological advantages of self-generated electricity. Commercially available PV panels made from monocrystalline silicon typically achieve a solar-to-electric energy conversion efficiency of 18–22%. The theoretical physical limit for silicon-based solar cells is approximately 29%, representing the upper boundary that current research continues to approach.

The integration of PV systems with heating technologies, including underfloor heating systems, has become an active field of study. Using PV panels for underfloor heating brings both benefits and limitations. The challenges include the mismatch between solar availability and heating demand, especially in winter, which often necessitates costly storage or backup systems [26]. High upfront investment, space requirements for panels, and potential efficiency losses in cloudy or cold conditions further limit standalone use. For this reason, solar-powered floor heating is most effective when combined with storage or auxiliary heating in a hybrid system. On the positive side, it provides a renewable and sustainable heat source that reduces reliance on fossil fuels, lowers energy bills, and supports long-term energy independence [27]. Underfloor heating systems operate efficiently at low temperatures, making it well-suited for solar integration, and once installed, the system has low operating costs and minimal maintenance. Recent studies show that hybrid systems combining solar heating with radiant floor systems can achieve up to 93% reduction in heating demand, demonstrating their potential for zero-energy buildings [28,29]. Potential areas for further research include smart control systems that coordinate electricity production with heating demands, battery storage solutions, and the utilization of building structures’ thermal inertia. Equally important is the integration of hybrid solutions and long-term evaluation of both environmental and economic impacts.

The objective of this study is to assess, through numerical modeling, the efficiency of underfloor heating based on the amount and type of electricity supplied, while also examining the potential of solar panels. The efficiency of underfloor heating systems is assessed based on the quantity and type of electricity supplied, while the potential integration of solar panels is explored through numerical modeling. A sensor-driven approach to enhancing thermal efficiency and energy sustainability in residential heating is presented. A hybrid configuration is investigated, in which real-time solar radiation data and localized outdoor temperature measurements are utilized to optimize indoor thermal comfort. System performance is evaluated through numerical modeling and simulation under three energy supply scenarios—standard grid, solar-only, and hybrid—and the role of sensor data in intelligent energy management is highlighted.

The following sections outline the numerical testing methodology and the evaluation of selected underfloor heating modes in a test room. The results include the time evolution of indoor air temperature, speed of reaching equilibrium (determined via the time constant), heat loss through the external wall, and distribution of temperature and humidity at equilibrium. Specific findings are discussed, and general conclusions are drawn in the final section.

## 2. Materials and Methods

The study based on numerical analysis included the following steps:

- Compilation of a graphic drawing of the monitored room model.
- Defining the thermophysical properties of the individual components of the model.
- Insertion of the initial conditions of the boundary conditions of the observed event.
- Numerical simulations using COMSOL Multiphysics version 6.3 (COMSOL, Stockholm, Sweden).
- Export of the required output data, their subsequent processing, and detailed analytical evaluation by the MATLAB version 2025a (MathWorks, Natick, MA, USA).

The applicability of COMSOL Multiphysics computational tools was validated in the previous study [30] through a comparative Building Energy Simulation Test [31].

A detailed description of the assembled model, including its geometry, material properties of individual components, and simulation conditions within the COMSOL environment, is stored at the IEEE DataPort https://dx.doi.org/10.21227/dhs3-at84 (accessed on 1 September 2025) [32]. This repository also contains a video abstract of the paper and the output dataset from the simulations.

**Figure 1:**
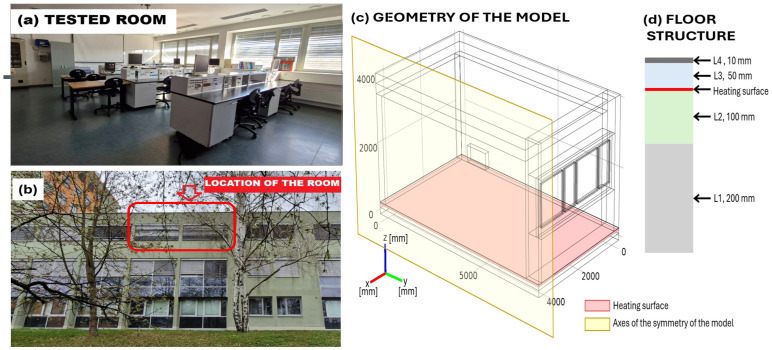
Tested room and its model. (**a**) Photo of the room interior. (**b**) Outside view of the room location. (**c**) Half-room geometry and symmetry plane display. (**d**) Structure of the model’s floor.

The assessed object is a room shown in Figure 1a. It is located under the roof of the building and is surrounded on both sides by neighboring rooms; a corridor in the interior of the building is depicted in Figure 1b.

The geometry of the model of the room under study is shown in Figure 1c,d. A heating surface was placed in the floor as is depicted in Figure 1d. To eliminate unwanted heat flow to the lower layer of the floor, a thermal insulation of 10 cm thickness was placed under the heating layer. A condition of symmetry was used for a model of half the room, as shown in Figure 1c, to save computational time and computer memory. This condition includes symmetry in the geometry as well as the physical conditions of the left and right halves of the room. The thermophysical properties of the materials used to formulate the model are summarized in Table 1.

Heating simulations were performed assuming that the initial air temperature in the room was 10 °C. The air temperature in the surrounding rooms was 15 °C. The outdoor air temperature of 0 °C represents an average value calculated from data measured at the location of the building during the monitored period.

**Figure 2:**
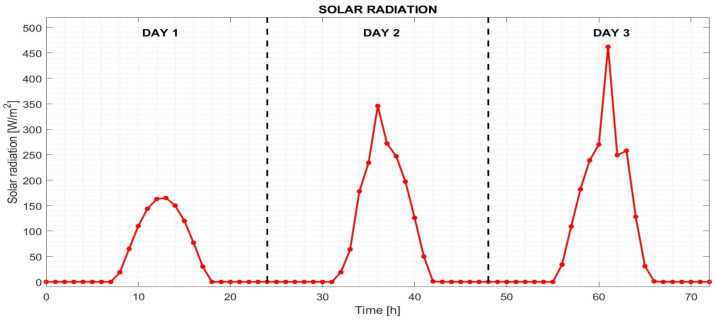
Boundary condition of simulations indicating the intensity of solar radiation during three days of room heating.

The values of solar radiation intensity on the monitored days are depicted in Figure 2. The values were obtained from meteorological data in the Central Europe location in the first half of March. A heat transfer coefficient on the outside of the building was 23 $W/\left(m^{2}\cdot K\right)$, heat transfer coefficients inside the building were 8 $W/\left(m^{2}\cdot K\right)$ and 6 $W/\left(m^{2}\cdot K\right)$. These values are recommended by the Czech technical standard addressing the issue of thermal protection of buildings [33].

Numerical calculations in COMSOL Multiphysics included nonstationary heat transfer by conduction, convection, and radiation in the room during the selected time period of 72 h. A physical description used to solve the problem can be found in the publication [34].

The transient heat transfer in a building envelope is governed by the heat conduction equation, which incorporates spatial and temporal temperature variations (1) [35,36]:

(1) $$\nabla \left(-k\nabla T\right)+\varrho c_{p}v\nabla T+\varrho c_{p}\frac{\partial T}{\partial t}=\Phi ,$$

where *k* is the thermal conductivity, $\left[W/\left(m\cdot K\right)\right]$, ϱ is the density, $\left[\mathrm{kg}/m^{3}\right]$, $c_{p}$ is the specific heat capacity, $\left[J/\left(\mathrm{kg}\cdot K\right)\right]$, *T* is the thermodynamical temperature, $\left[K\right]$, *v* is the fluid velocity, $\left[m/s\right]$, and Φ is the inner heat-generation rate per unit volume, $\left[W/m^{3}\right]$.

Heat transfer between the surface of solid building materials and ambient air is characterized by the boundary condition for heat flux density

(2) $$q_{s}=h\left(\theta_{i}-\theta_{si}\right),$$

where $q_{s}$ is the heat flux density, $\left[W/m^{2}\right]$, *h* is the convective heat transfer coefficient, $\left[W/\left(m^{2}\cdot K\right)\right]$, $\theta_{i}$ is the fluid temperature, [°C], and $\theta_{si}$ is the temperature of the surface of the solid material, [°C].

A radiative heat transfer condition was applied to the boundaries adjacent to the internal air, and is mathematically expressed as

(3) $$q_{r}=\varepsilon \sigma \left(T_{si}^{4}-T_{amb}^{4}\right),$$

where ε is the emissivity, [−], $T_{si}$ is the boundary thermodynamical temperature, $\left[K\right]$, σ is the Stephan–Boltzmann constant, $\sigma =5.670367\cdot 10^{-8}\;W\cdot m^{-2}K^{-4}$, and $T_{amb}$ is the thermodynamical ambient temperature, $\left[K\right]$.

**Figure 3:**
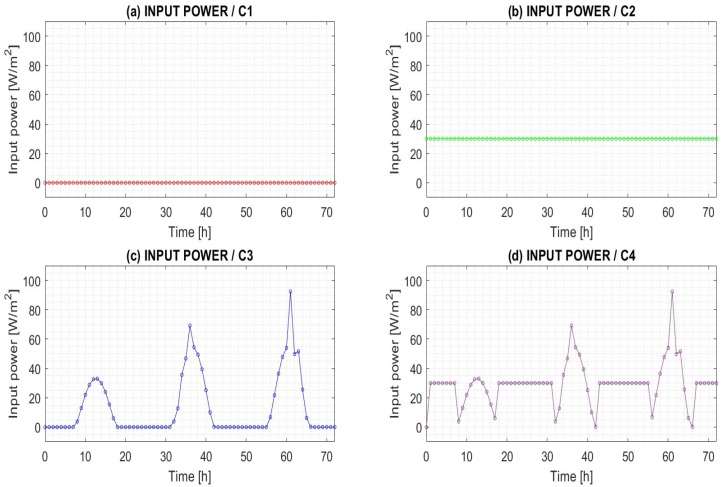
Input power used for underfloor heating. Studied cases (**a**) C1—no heating power; (**b**) C2—heating power 30 W/m^2^; (**c**) C3—heating power is 20 percent of the current solar radiation; (**d**) C4—a combination of 20 percent of current solar radiation and 30 W/m^2^ of supplied energy.

Depending on the amount of energy used for underfloor heating, the following cases were solved:

- Case 1 (C1)—No heating power (see Figure 3a).
- Case 2 (C2)—Heating power 30 W/m^2^ (see Figure 3b).
- Case 3 (C3)—Heating power is provided by monocrystalline photovoltaic panels, which are commonly used under the environmental conditions of the test site. An assumed solar-to-electric conversion efficiency of approximately 20 percent was applied (see Figure 3c).
- Case 4 (C4)—A combination of 20 percent of current solar radiation and 30 W/m^2^ of supplied energy (see Figure 3d).

The total amount of electricity used for heating over a 72 h period and the proportion of electricity supplied by PV panels is summarized in Table 2.

The efficiency of the given heating modes was compared by determining the time constant defined as the time required for a system’s response to reach approximately 63.2% of steady interior air temperature [37]. In the present study, the time constant was calculated as the time value to reach an increase in air mean temperature from the initial value to the value of 63.2% of the final temperature, i.e., to the temperature reached after 72 h of room heating. To determine the time constant value, the air mean temperature datasets were approximated by third-order polynomial functions in Case C1, and fifth-order polynomial functions in cases C2, C3, and C4 by using the Curve Fitter computational tool of MATLAB software.

To verify the suitability of the monitored heating method for a given room, an assessment was also carried out in terms of the moisture content of the external wall according to the procedure given by the Czech Technical Standard for Construction and is based on determining the distribution of water vapor pressures in a typical location of the structure by use the Teplo 2017 software (K-CAD, Prague, Czech Republic), which is intended for basic thermal technical assessment of the building structure in terms of heat and water vapor transmission.

## 3. Results

The effectiveness of the monitored underfloor heating modes was assessed by comparing the room air temperature and the heat flow through the external wall over a three-day (72 h) period, under the climatic conditions described in the previous section. The exported dataset is stored at the IEEE DataPort https://dx.doi.org/10.21227/dhs3-at84 (accessed on 1 September 2025) [32] for further investigation.

### 3.1. Efficiency Comparison of Different Room Heating Modes

**Figure 4:**
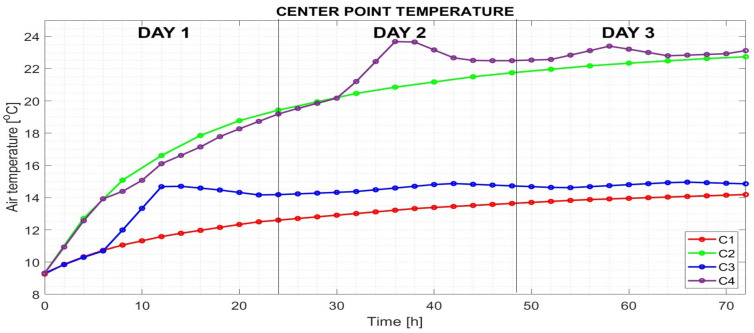
Time evolution of the air temperature at the center point of the heated room in studied cases C1, C2, C3, and C4.

The output data from computer simulations, illustrating the time evolution of air temperature at the center of the room, are presented in Figure 4. Heating modes C2 to C4 were evaluated using a 2 h time step and compared with the unheated room model C1, in which the air temperature was influenced solely by the temperatures of adjacent rooms and the outdoor environment. After 72 h, the air temperature in model C1 reached 14.2 °C.

For mode C2, with a constant heating power of 30 W/m^2^ (see Figure 3b), the air temperature at the center of the room reached 22.7 °C after 72 h of heating. In contrast, under mode C3, where only solar radiation was used for heating (as shown in Figure 3c), daily fluctuations in solar radiation led to corresponding variations in room air temperature. Over the 72 h period, the air temperature at the room’s center was calculated to be 14.9 °C.

In heating mode C4, energy was supplied in a combined manner: a constant heating power of 30 W/m^2^ was maintained during the night, while solar radiation was used for heating during the day, following the time course shown in Figure 3d. Similar to mode C3, mode C4 exhibited fluctuations in room air temperature. After 72 h of heating, the air temperature at the center of the room reached 23.1 °C.

**Figure 5:**
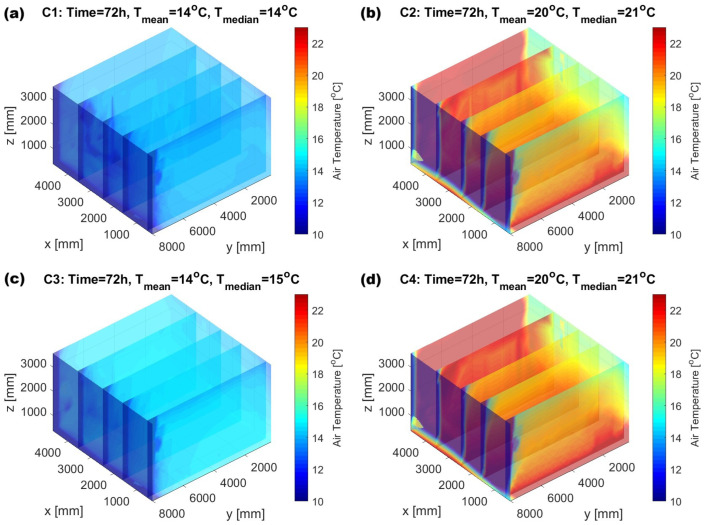
Distribution of air temperature inside the room at 72 h for heating modes (**a**) C1, (**b**) C2, (**c**) C3, and (**d**) C4.

**Figure 6:**
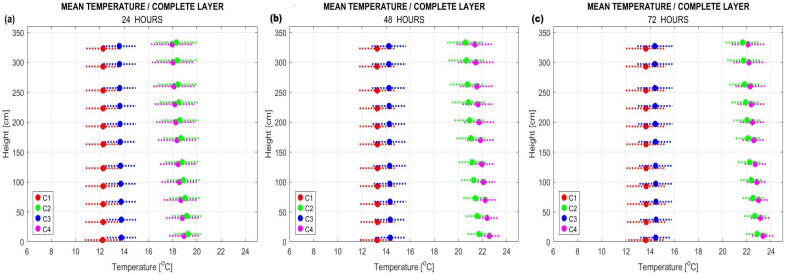
Comparison of mean air temperatures at selected vertical (z-axis) positions in the room at (**a**) 24 h, (**b**) 48 h, and (**c**) 72 h for heating modes (**a**) C1, (**b**) C2, (**c**) C3, and (**d**) C4.

The visualization of air temperature distribution throughout the room after 72 h of heating is presented in Figure 5. In cases C2, C3, and C4, the impact of underfloor heating on vertical air temperature gradients—from floor to ceiling—is clearly observable. However, this variation does not significantly affect the mean air temperature values, as shown in Figure 6, where mean temperatures were calculated for selected height zones (i.e., distances from the floor) at 24, 48, and 72 h.

**Figure 7:**
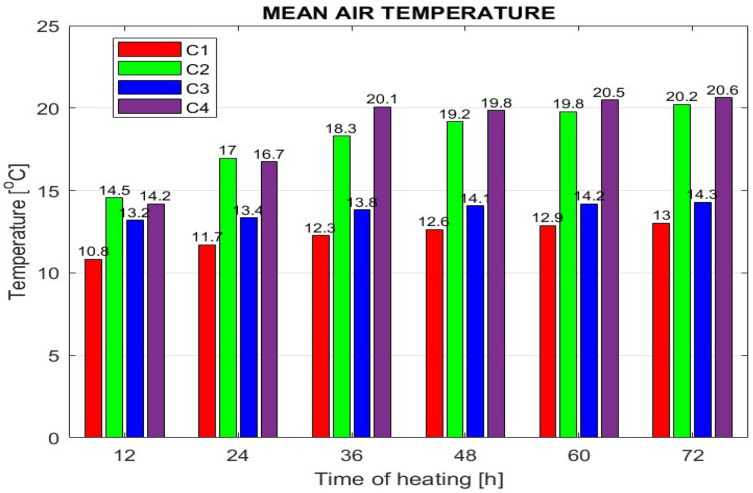
Air mean temperature at the selected room heating time.

The efficiency of the monitored heating modes was further evaluated based on mean room temperature values, which were calculated by MATLAB after processing and approximating the simulation output data. The resulting mean air temperatures over the heating period from 12 to 72 h are graphically represented in Figure 7.

**Figure 8:**
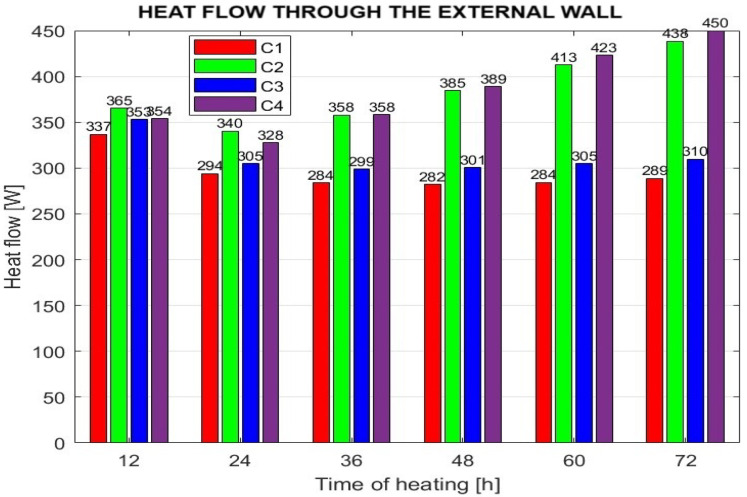
Heat flow through the external wall at the selected room heating time.

During the heating period from 12 to 72 h, heat losses through the room’s exterior wall were also monitored. The results are presented in Figure 8.

The graph compares the mean values of approximated data representing heat flow across the outer surface of the exterior wall, including windows and window frames. Only the component of heat flow perpendicular to the vertical wall was evaluated. The results indicate that the trend in heat flow values corresponds to the temperature difference between the interior and exterior of the building.

As described in the Methods section, efficiency of the assessed heating modes was also compared based on the time constant, which represents the time required to reach 63.2% of the final room air temperature. The calculated time constant values are summarized in Table 3. In case C1, where the room was heated solely through heat transfer from adjacent rooms, the time constant was the highest—measured at 28.8 h. In contrast, for the heating mode C3, the time constant was significantly lower, at 8.5 h.

### 3.2. Assessment of External Wall Moisture Risk

**Figure 9:**
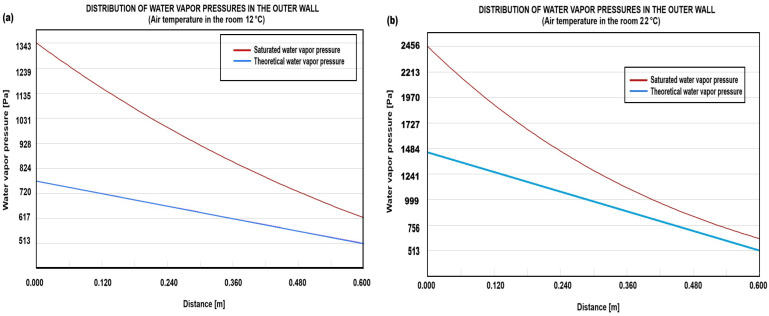
Water vapor pressure distribution across the exterior wall section from outside to inside, under two indoor temperature scenarios: (**a**) 12 °C and (**b**) 22 °C. The outdoor air temperature is fixed at 0 °C.

The moisture content of the external wall was assessed by analyzing the distribution of water vapor pressures at a representative location within the structure using the Teplo 2017 software. The results indicate that no moisture condensation occurs within the wall when the room air temperature ranges between 12 °C and 22 °C. A graphical representation of the water vapor pressure distribution in the wall, corresponding to an indoor air temperature of 12 °C, is shown in Figure 9a. Similarly, Figure 9b presents the distribution of water vapor pressures under the same indoor temperature conditions.

## 4. Discussion

The findings from the computer simulations provide valuable insights into the effectiveness of various underfloor heating modes in maintaining optimal room air temperature and minimizing heat loss. The results indicate that heating power and energy sourcing significantly influence thermal behavior over time.

**Figure 10:**
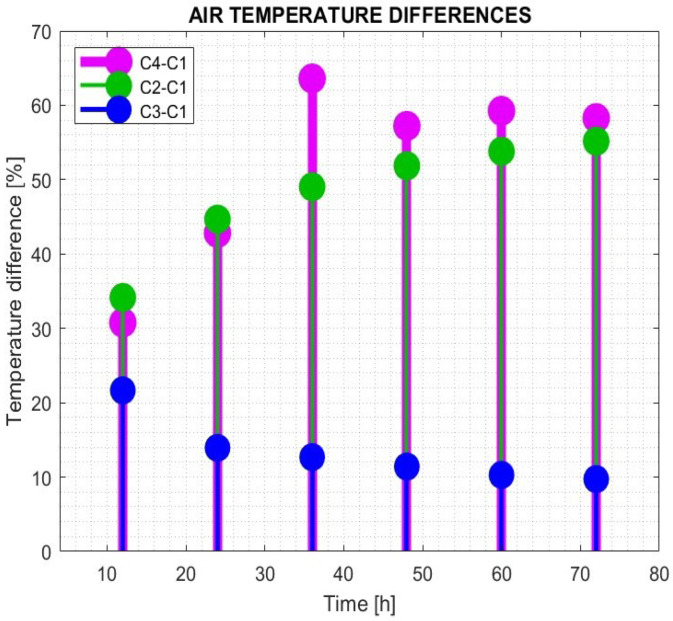
Air temperature differences between heated rooms C2, C3, C4, and unheated room C1.

The analysis of air temperature at the center of the room demonstrates that different heating approaches lead to varying thermal stability. The absence of underfloor heating in case C1 results in a relatively low temperature increase over the monitored period of 72 h. The air temperature differences in C2, C3, and C4, relative to the unheated room C1, were calculated for selected heating times and are displayed in Figure 10.

When a constant heating power of 30 W/m^2^ was applied in C2, the temperature differences ranged between 34.14% and 55.2% compared to C1 at the monitored times. The contribution of solar radiation alone in C3 resulted in a temperature increase of between 9.7% and 21.7% relative to C1. The combined approach in C4 yielded the highest efficiency, achieving a temperature difference of between 30.8% and 63.6% compared to the unheated room C1.

As shown in Table 2, under the specified conditions for case C4, approximately 50% of the total electrical energy used for heating was supplied by PV panels. This outcome is favorable from both economic and environmental standpoints, as it demonstrates the potential for reducing reliance on grid electricity and lowering associated emissions. Moreover, it provides a preliminary insight into the contribution of PV-generated energy across different heating scenarios, supporting the feasibility of integrated renewable energy systems in residential applications.

To further evaluate heating efficiency, the mean air temperatures from 12 to 72 h were calculated using MATLAB, providing a comprehensive comparison between the different heating strategies. Additionally, the time constant analysis reveals notable differences in thermal response among the cases. Case C1 exhibited the longest stabilization time, reaching 28.8 h. In contrast, cases C2 and C4, which employed continuously supplied heating power, achieved stabilization within 22 h. In the case of C3, when only solar energy was supplied intermittently, the stabilization time was 8.5 h.

**Figure 11:**
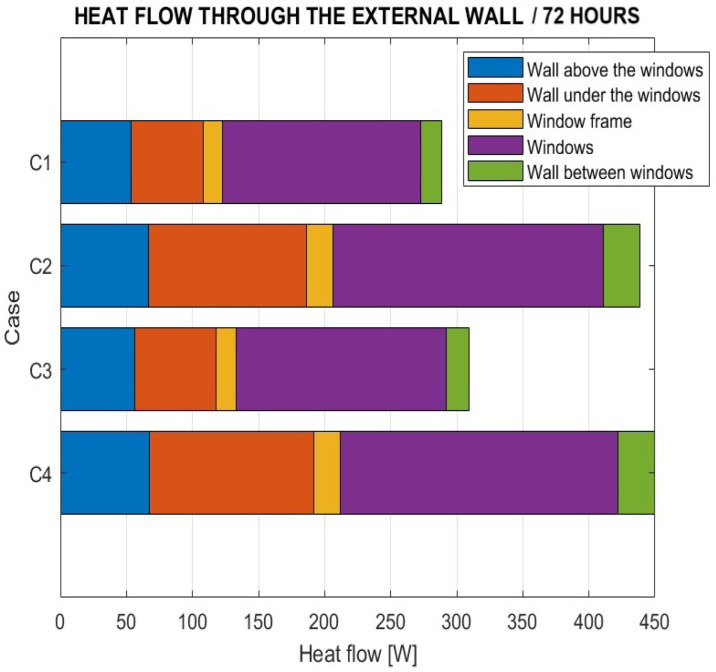
Heat flow through the external wall after 72 h of heating the room.

The study also investigated heat loss through the external wall, revealing a correlation with the air temperature differences between the interior and exterior of the room. Figure 11 compares heat flow through various segments of the external wall for cases C1, C2, C3, and C4 at the 72 h mark. The windows and the wall section below them were identified as the most significant areas of heat loss. The proportion of heat loss through the windows relative to the total heat loss through the external wall was approximately 51% in case C3 and 52% in case C1. In cases C2 and C4, this proportion was around 47%. Heat loss through the wall below the windows accounted for approximately 28% in case C4, 27% in case C2, 20% in case C3, and 19% in case C1. Based on these findings, recommended modifications to the existing building include mainly the application of appropriate thermal insulation to the perimeter walls and replacing existing windows with models featuring lower thermal transmittance.

The analysis of water vapor pressure distribution within the external wall demonstrates the effectiveness of the monitored heating method in preventing moisture condensation. The findings indicate that across the evaluated temperature range of 12 °C to 22 °C, no condensation occurs, which suggests that the internal thermal environment remains within acceptable limits to mitigate excess humidity accumulation. This outcome is significant as condensation within building structures can lead to material degradation, mold formation, and a decline in thermal insulation performance. The graphical representation provided in Figure 9a,b further illustrates the consistency of vapor pressure distribution, reinforcing the conclusion that moisture-related risks are negligible under the given conditions. Furthermore, these results align with the assessment procedures outlined in the Czech Technical Standard for Construction, confirming that the methodology used for evaluating the heating system is robust and reliable. The absence of condensation within the wall indicates that the heating system maintains stable indoor air parameters without inducing excessive moisture accumulation.

Although the present study relies primarily on numerical simulations, the integration of sensor-based experimental validation would significantly enhance the robustness and credibility of the findings. A distributed network of sensors could be deployed within the test room to capture critical thermal and environmental parameters. For instance, temperature sensors placed at various heights—from near the floor through mid-room to the ceiling—would facilitate quantification of vertical temperature gradients and enhance the fidelity of comfort assessments [38].

Humidity sensors, positioned both in the indoor environment and within wall layers, would support the evaluation of condensation risk, complementing the vapor pressure analysis performed in simulation [39,40]. Heat flux sensors installed on external walls and windows could provide direct measurements of heat loss, while pyranometers would allow accurate recording of incident solar radiation for correlation with PV-based heating scenarios. Smart energy meters would complete the measurement framework by tracking the real-time electricity consumption of the heating system. Together, these sensing modalities would establish a comprehensive dataset for model validation, adaptive control strategies, and long-term monitoring of energy efficiency and thermal comfort. Similar sensor-based studies of radiant floor heating systems have been reported in [41], confirming the feasibility of integrating simulation with experimental data acquisition.

This study was limited to simulations conducted within the COMSOL Multiphysics software. Due to the high computational memory demands, the 3D room model had to be simplified in terms of its geometric configuration and boundary conditions, including the omission of occupancy-related conditions, the use of fixed heat transfer coefficients, and the neglect of infiltration effects. For comprehensive assessments of whole-building energy performance or long-term energy balance studies, specialized simulation tools such as EnergyPlus or TRNSYS are necessary.

## 5. Conclusions

In this study, numerical simulations were employed to evaluate underfloor heating performance using energy supplied by the standard electrical grid, solar-generated energy, and a combined configuration. It was primarily focused on the methodology for evaluating the efficiency of selected underfloor heating modes, based on the analysis of the air temperature time evolution in simple room models. The findings confirm that the combined heating mode C4 is the most effective for maintaining a stable and warm indoor environment, efficiently utilizing both solar radiation and supplementary energy sources. Under the conditions considered in this study, the indoor air temperature increased by up to 63.6% compared to an unheated room.

The heat loss assessment revealed the main locations of heat loss in the room and for the design of possible building modifications.

The assessment of the risk of wall dampness confirmed that during all tested heating regimes, there is no condensation of water vapor within the walls of the room.

Future work should integrate detailed thermal modeling with whole-building energy analysis to enhance simulation accuracy and scope. The investigations could explore further optimizations, including seasonal variations in solar input and alternative energy-efficient heating strategies. Also, the influence of special materials, such as phase change material, in radiant underfloor heating systems could be tested.

The integration of advanced sensor systems could enable real-time monitoring and adaptive control of hybrid heating systems, further enhancing their performance. Environmental sensors could precisely evaluate the thermal behavior of such materials under varying conditions, contributing to more efficient designs.

In terms of building walls’ wetting risk prevention, future studies could explore variations in external climate conditions, such as colder temperatures or elevated humidity levels, to assess potential changes in vapor pressure distribution. High-resolution humidity and temperature sensors could help map these variations and their impact more accurately. Furthermore, long-term monitoring could provide further insights into the seasonal impact on moisture accumulation and structural integrity, with sensors enabling continuous data collection and analysis.

## Figures and Tables

**Table 1:** Material properties of the model elements.

Material	Thermal Conductivity [W/(m·K)]	Density [kg/m^3^]	Specific Heat Capacity [J/(kg·K)]	Emissivity [−]
Front wall	0.27	900	960	0.94
Structure in the front wall	1.43	2300	1020	0.94
Side wall	0.27	900	960	0.94
Back wall	0.27	900	960	0.94
Lower layer of the ceiling	0.039	30	1270	0.94
Upper layer of the ceiling	1.43	2300	1020	-
Floor-L1	1.34	2400	840	-
Floor-L2 (therm. insulation)	0.039	30	1550	-
Floor-L3	1.1	2200	840	-
Floor-L4 (floor tiles)	1.0	840	2000	0.90
Window frame	0.18	400	2510	0.85
Window glass	1.34	2400	840	0.92

**Table 2:** Amount of electricity supplied for the room heating over a 72 h period.

Heating Mode	Grid Electricity [kWh]	PV Panels Energy [kWh]	Total Supplied Energy [kWh]
C1	0	0	0
C2	68.8	0	68.8
C3	0	36.4	36.4
C4	37.3	37.4	74.7

**Table 3:** Time constant of the heated room.

Heating mode:	C1	C2	C3	C4
Time constant [h]:	28.8	22.2	8.5	22.0

## Data Availability

The output dataset from the computer simulations and video abstract of the paper are stored at the IEEE DataPort https://dx.doi.org/10.21227/dhs3-at84 (accessed on 1 September 2025).
